# 埃克替尼在晚期非小细胞肺癌EGFR状态明确的患者中的疗效分析

**DOI:** 10.3779/j.issn.1009-3419.2013.03.04

**Published:** 2013-03-20

**Authors:** 正波 宋, 新民 余, 菊芬 蔡, 岚 邵, 宝钗 林, 春晓 何, 贝贝 张, 沂平 张

**Affiliations:** 1 310022 杭州，浙江省肿瘤医院化疗中心 Department of Chemotherapy, Zhejiang Cancer Hospital, Hangzhou 310022, China; 2 310022 杭州，浙江省胸部肿瘤诊治技术重点实验室 Key Laboratory Diagnosis and Treatment Technology on Thoracic Oncology, Zhejiang Province, Hangzhou 310022, China

**Keywords:** 肺肿瘤, 埃克替尼, *EGFR*突变, 疗效, Lung neoplasms, Icotinib, *EGFR* mutation, Efficacy

## Abstract

**背景与目的:**

埃克替尼是国内第一个口服的表皮生长因子受体（epidermal growth factor receptor, EGFR）酪氨酸激酶受体抑制剂，在体内外实验研究中显示出对非小细胞肺癌的明显抑制作用。Ⅲ期临床研究ICOGEN显示埃克替尼对复治晚期非小细胞肺癌疗效不劣于吉非替尼。本研究探讨在晚期非小细胞肺癌明确EGFR状态的患者中（*EGFR*野生型和突变型）埃克替尼的疗效和安全性。

**方法:**

回顾性分析2011年8月-2012年8月在浙江省肿瘤医院就诊并行埃克替尼治疗的晚期非小细胞肺癌患者，*Kaplan*-*Meier*法进行生存分析和比较。

**结果:**

49例患者明确了*EGFR*突变状态并行埃克替尼治疗，49例患者中13例为野生型，36例为突变型。突变患者的客观缓解率和疾病控制率分别为58.3%和88.9%，野生型患者的客观缓解率和疾病控制率分别为7.7%和53.8%。突变和野生型患者的中位无进展生存期为9.5个月和2.2个月（*P* < 0.001）。36例突变患者中一线治疗19例，二线及二线以上患者17例。一线和复治患者的中位无进展生存期（progression-free survival, PFS）分别为9.5个月和8.5个月（*P*=0.41）。突变型患者的中位总生存期（overall survival, OS）尚未达到，野生型患者的OS为12.6个月。患者的不良反应以皮疹和腹泻为主，但多为轻到中度。

**结论:**

埃克替尼在*EGFR*突变患者中的疗效较好，可以作为*EGFR*突变患者的优选方案。患者的毒副反应多数可以耐受。

## Abstract (en)

**Background and objective:**

As the first epidermal growth factor receptor tyrosine kinase inhibitor (EGFR-TKI) in China, icotinib shows promising anticancer activity *in vitro* and *vivo*. The phase Ⅲ clinical study (ICOGEN) showed that icotinib has a good efficacy and tolerability in Chinese patients with advanced non-small cell lung cancer (NSCLC) compared with gefitinib. This retrospective study aims to evaluate the ef?cacy and tolerability of icotinib monotherapy for advanced NSCLC patients with *EGFR* mutation and wild-type patients in our hospital.

**Methods:**

Patients with advanced NSCLC who were treated with icotinib in Zhejiang Cancer Hospital were retrospectively analyzed from August, 2011 to August, 2012. Survival was estimated using *Kaplan*-*Meier* analysis and *Log*-*rank* tests.

**Results:**

The clinical data of 49 patients (13 with wild-type and 36 with *EGFR* mutation) with NSCLC were enrolled in the current study. The patients' overall objective response rate (ORR) was 58.3% and the disease control rate (DCR) in 36 *EGFR* mutation patients was 88.9%. The ORR was 7.7% and DCR was 53.8% in the wild-type patients. Median progression-free survival (PFS) with icotinib treatment in *EGFR* mutation patients was 9.5 months and 2.2 months in wild-type patients (*P* < 0.001). Nineteen patients with *EGFR* mutation received icotinib as first-line and 17 in further-line treatment. The PFS was 9.5 months in the first-line and 8.5 months for second-line or further-line patients (*P*=0.41). Median overall survival (OS) in *EGFR* mutation patients was not reached, but was 12.6 months in wild-type patients. Most of the drug-related adverse events were mild (grade Ⅰ or Ⅱ) and reversible with no grade Ⅳ toxicity.

**Conclusion:**

Icotinib monotherapy showed significant antitumor activity in advanced NSCLC *EGFR* mutation patients. The toxicity was well tolerated and acceptable.

非小细胞肺癌（non-small cell lung cancer, NSCLC）是目前发病和死亡第一位的肿瘤，严重威胁人类的生存^[1]^。其中晚期NSCLC约占患者总数的60%以上^[2]^。传统的晚期NSCLC的治疗以化疗为主，但疗效有限，患者的中位生存时间约10个月左右^[3]^。靶向治疗的问世不仅使部分患者的生存期提高，而且大大改善了生活质量。吉非替尼和厄洛替尼是口服的酪氨酸激酶受体抑制剂（tyrosine kinase inhibitor, TKI)，能明显提高具有表皮生长因子受体（epidermal growth factor receptor, EGFR）突变患者的疗效，其中位生存时间在EGFR突变患者中已经超过两年^[4, 5]^。埃克替尼是我国自主研发的第一个EGFR-TKI药物，也是继吉非替尼和厄洛替尼后第三个EGFR-TKI药物。ICOGEN^[6]^是第一个头对头比较埃克替尼与吉非替尼疗效的Ⅲ期临床研究。结果显示，两药疗效相近，但埃克替尼毒副反应低于吉非替尼。该研究共入组399例患者，其中仅29例具有EGFR突变的患者服用埃克替尼，因此，对于埃克替尼在EGFR突变患者中的疗效还需要进一步验证。本研究旨在回顾性分析浙江省肿瘤医院行EGFR突变检测并明确突变状态的患者服用埃克替尼的疗效和安全性，为临床用药提供参考。

## 1 资料与方法

### 1.1 临床资料

2011年8月-2012年8月在浙江省肿瘤医院就诊的具有完整随访资料的Ⅲb期和Ⅳ期晚期NSCLC患者，共221例患者使用了埃克替尼治疗。本研究排除标准包括：①患者EGFR突变状况未知；②无可测量的临床病灶；③服用埃克替尼过程中伴有其它可能影响患者疗效评价的治疗手段，如放疗、介入等；④患者ECOG评分>3分。

### 1.2 疗效评价

患者在埃克替尼治疗开始1个月后评估疗效，疗效稳定或有效患者每两个月接受一次CT和其它影像学检查进行疗效评价。根据RECIST 1.1标准进行疗效评价，分为完全缓解（complete response, CR）、部分缓解（partial response, PR）、疾病稳定（stable disease, SD）、疾病进展（progressive disease, PD）。近期有效率包括完全缓解和部分缓解（response rate, RR）。疾病控制率为完全缓解，部分缓解和稳定（CR+PR+SD, DCR）。

### 1.3 毒副反应

按照美国NCI制定的毒副反应标准（CTC第3版）评价毒副反应，分为0-4共5个等级。

### 1.4 患者*EGFR*突变检测方法

采用焦膦酸测序法检测患者的*EGFR*突变状态，检测标本包括15例手术标本，25例活检标本，9例其它组织标本。

### 1.5 随访

49例患者均获得随访，患者无进展生存期（progression-free survival, PFS）通过门诊或电话随访获得，中位随访时间为10.1个月，末次随访时间为2012年12月20日。总生存期（overall survival, OS）指患者自首次治疗起到患者死亡或末次随访的时间。PFS定义为患者自埃克替尼开始到明确疾病进展的时间。

### 1.6 统计学分析

采用SPSS17.0统计软件进行统计分析，*Kaplane*-*Meier*法分析患者中位PFS及OS，*P* < 0.05为差异具有统计学意义。

## 2 结果

### 2.1 患者的一般特征

共49例患者纳入本研究。其中包括26例男性，23例女性。所有患者的中位年龄为57岁。44例患者病理类型为腺癌，5例为其它病理类型。Ⅲb期患者共4例，其余均为Ⅳ期患者。49例患者中包括13例EGFR野生型，36例EGFR突变型。36例突变类型中，18例为19外显子缺失，15例为外显子21 L858R突变，其它突变类型3例。患者的一般特征见表 1。

**1:** 49例患者的一般特征 Baseline characteristics of the study population (*n*=49)

Variables	All (*n*=49)	*EGFR* mutation (*n*=36)	EGFR wild-type (*n*=13)	*P*
Gender				0.17
Male	26	17	9	
Female	23	19	4	
Performance status				0.88
0-1	31	23	8	
2	18	13	5	
Age (year)				0.99
Median	57	57	58	
< 65	34	25	9	
≥65	15	11	4	
Smoking characteristics				0.41
Yes	18	12	6	
No	31	24	7	
Stage				0.94
Ⅲb	4	3	1	
Ⅳ	45	33	12	
Histology				0.83
Adenocarcinoma	44	34	10	
Non-adenocarcinoma	5	2	3	
EGFR: epidermal growth factor receptor.				

### 2.2 临床疗效评价

36例EGFR突变患者中CR患者0例，PR患者21例，SD患者11例，PD患者4例，客观缓解率为58.3%，疾病控制率为88. 9%。13例野生型患者中PR 1例，SD 6例，PD 6例，客观缓解率为7.7%，疾病控制率为53.8%（表 2）。

**2:** *EGFR*突变与野生型患者的疗效比较 The efficacy of icotinib in *EGFR* mutation and wild-type patients

Best response	Mutation [*n* (%)]	Wild-type [*n* (%)]	*P*
Complete response	0 (0)	0 (0)	-
Partial response	21 (17.7)	1 (11.4)	-
Stable disease	11 (30.6)	6 (43.1)	-
Progressive disease	4 (51.6)	6 (45.5)	-
Response rate	58.30%	7.70%	0.001, 7
Disease control rate	88.90%	53.80%	0.007, 2
Median progression-free survival (month)	9.5	2.2	< 0.001
Median overall survival (month)	-	12.6	< 0.001

### 2.3 PFS及总生存期分析

49例患者中位PFS为8.5个月，其中突变患者的中位PFS为9.5个月，外显子19突变患者的中位PFS为11.0个月，外显子21 L858R突变患者的中位PFS为8.7个月，两者PFS时间相比无统计学差异（*P*=0.138）（图 1，表 3）。野生型患者的中位PFS为2.2个月，与突变患者的PFS相比有统计学差异（*P* < 0.001）（图 2）。突变患者中，12例脑转移患者的PFS为8.7个月，24例无脑转移患者的中位PFS为10.0个月，两者相比未见统计学差异（*P*=0.69）（图 3）。36例突变患者中一线治疗19例，二线及二线以上患者17例。一线和复治患者的PFS分别为9.5个月和8.5个月（*P*=0.41）。13例野生型患者中，7例取得疾病控制的患者的中位PFS为5.5个月，6例进展患者的中位PFS为1.2个月（*P* < 0.001）。截止到2012年12月20日，共21例患者死亡，其中突变患者14例，野生型患者7例。*EGFR*突变患者的中位OS尚未达到。13例野生型患者的OS时间为12.6个月。在野生型患者中取得疾病控制的患者与野生型患者的中位OS无统计学差异（*P*=0.057）。

**1:**
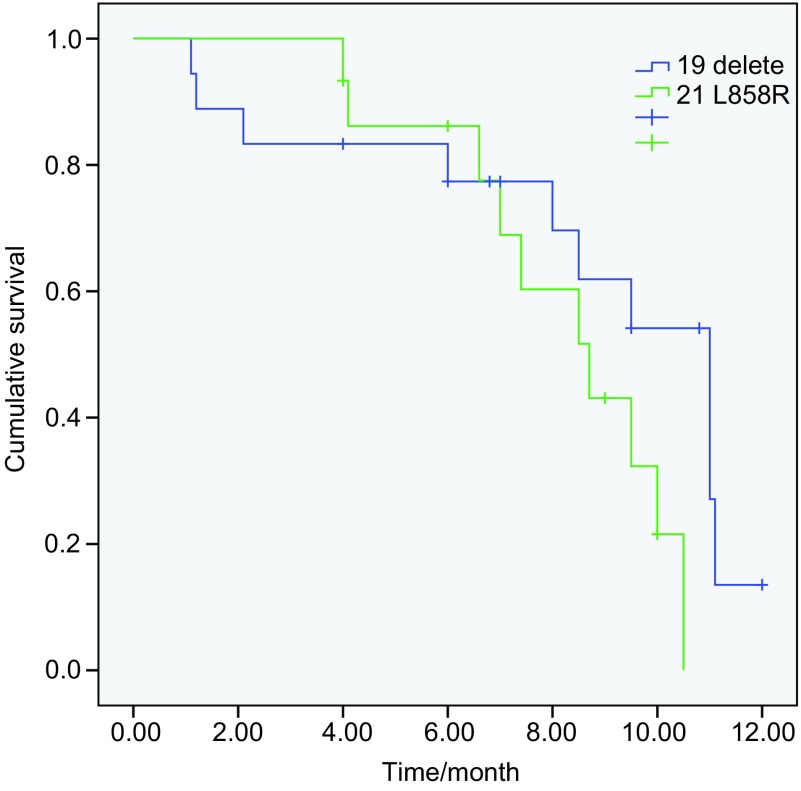
表皮生长因子受体外显子19缺失患者与外显子21 L858R突变患者的无进展生存期比较（11个月*vs* 8.7个月，*P*=0.138) Progression-free survival (PFS) in epidermal growth factor receptor (EGFR) exon 19 delete and exon 21 L858R mutation patients (11.0 months *vs* 8.7months, *P*=0.138)

**3:** 36例突变患者的单因素分析 Univariate analysis of PFS in 36 *EGFR* mutation patients

Variables	PFS	95%CI	*P*
Gender			0.39
Male	8.7	6.9-10.5	
Female	9.5	7.6-11.4	
Performance status			0.054
0-1	10.5	8.8-12.2	
2	7.0	5.8-8.2	
Age (year)			0.21
< 65	8.5	7.5-10.5	
≥65	10.8	9.6-12.0	
Smoking characteristics			0.75
Yes	8.7	5.6-11.8	
No	9.5	7.9-11.1	
Brain metastasis			0.69
Yes	8.7	7.0-9.9	
No	10.0	6.6-13.8	
Stage			0.22
Ⅲb	7, 4	8.0-11.0	
Ⅳ	9.5	-	
Prior chemotherapy			0.41
0	9.5	7.9-11.0	
≥1	8.5	6.5-10.5	
Mutation type			0.35
Exon 19	11.0	8.0-13.1	
Other	8.5	6.9-11.0	
PFS: progression-free survival.			

**2:**
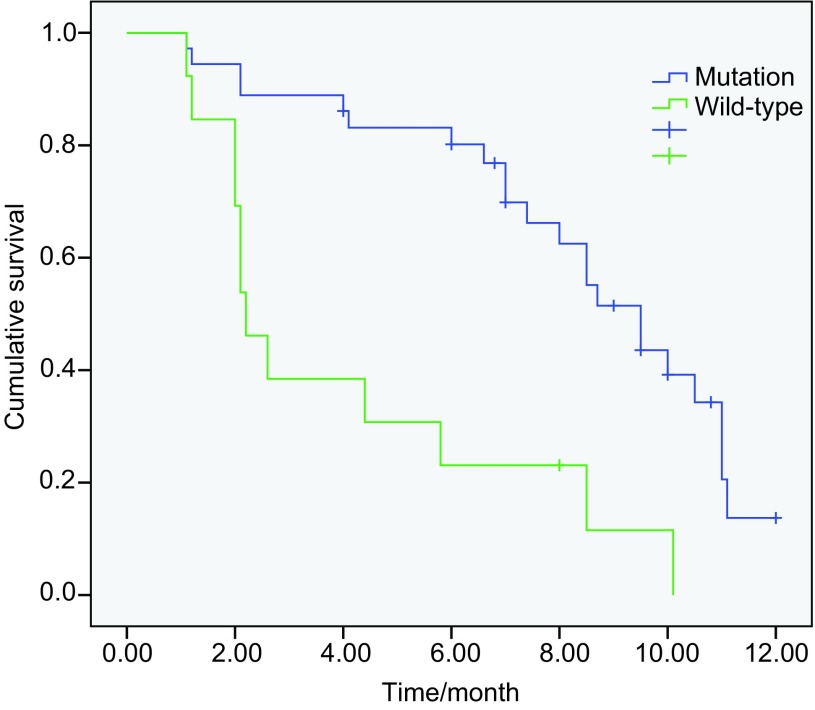
*EGFR*突变患者与野生型患者的PFS比较（9.5个月*vs* 2.2个月，*P* < 0.001） PFS in *EGFR* mutation and wild-type patients (9.5 months *vs* 2.2 months, *P* < 0.001)

**3:**
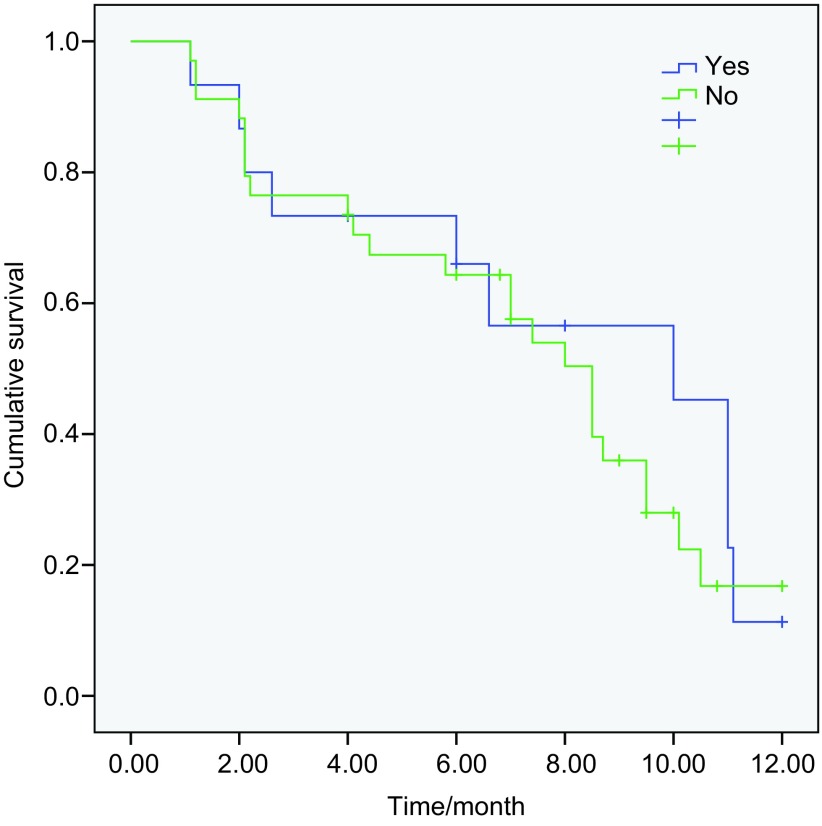
*EGFR*突变患者中脑转移与无脑转移患者的PFS比较（8.7个月*vs* 10.0个月，*P*=0.69) PFS of *EGFR* mutation patients with and without brain metastasis (8.7 months *vs* 10.0 months, *P*=0.69)

### 2.4 毒副反应

埃克替尼治疗过程中主要毒副反应为皮疹和腹泻；共19例患者发生皮疹反应（38.8%），腹泻发生率为24.5%（12/49）。Ⅲ度-Ⅳ度以上毒副反应发生率较低，所有患者总发生率为12.2%（6/49）（其中3例患者发生Ⅲ度皮疹，2例为Ⅲ度腹泻，1例为Ⅲ度疲乏），无Ⅳ度不良反应发生。

## 3 讨论

作为国内第一个也是继厄洛替尼和吉非替尼后国际上第3个EGFR-TKI药物，埃克替尼前期临床研究ICOGEN显示在复治晚期NSCLC中疗效与吉非替尼相当，但不良反应明显降低，目前已经成为国内复治晚期NSCLC治疗的标准药物之一。

本研究通过36例EGFR突变的患者服用埃克替尼的疗效分析表明，患者的中位PFS达到9.5个月，客观缓解率为58.3%，疾病控制率为88.9%，和既往报道^[7, 8]^吉非替尼疗效相似。这表明埃克替尼对EGFR突变患者的疗效较好。对13例野生型患者服用埃克替尼的疗效分析显示，患者的中位PFS为2.2个月，疾病控制率达到53.8%，这提示埃克替尼对部分野生型患者有一定的疗效。

对ICOGEN研究^[6]^EGFR突变的患者分析表明，埃克替尼组中位PFS为7.8个月。我们的研究中突变患者的PFS较ICOGEN研究的时间长，原因可能和本研究纳入的患者的PS评分相对较好有一定的关系。研究表明，在处方剂量下厄洛替尼（150 mg/d）能够抑制野生型细胞株的生长，而吉非替尼则无法抑制。埃克替尼前期的基础研究也显示对野生型细胞株疗效和厄洛替尼接近^[9]^。本研究13例EGFR野生型患者中位PFS为2.2个月，与TAILOR研究^[10]^的野生型患者2.4个月的中位PFS接近，这提示埃克替尼对野生型患者有一定的疗效。

外显子19缺失突变和外显子21 L858R突变是目前最主要的两种突变类型。有研究^[11]^显示，外显子19缺失突变患者服用EGFR-TKI的疗效好于外显子21 L858R突变患者，本研究结果同样提示外显子19缺失患者的疗效好于外显子21 L858R突变的患者（*P*=0.138），但未达到统计学差异，可能和本研究的样本量较小有关系。对于突变的患者，无论是一线还是在复治患者中使用，多数研究均显示疗效相近^[12]^，本研究对初治和复治突变患者的疗效分析同样显示没有疗效差异。有报道厄洛替尼和吉非替尼对于脑转移的患者疗效明显，特别是对于具有*EGFR*突变的患者^[13, 14]^。本研究中突变患者中共12例采用了埃克替尼治疗，中位PFS达到8.7个月，与既往报道相近，这表明对于*EGFR*突变的脑转移患者埃克替尼具有较好的疗效。安全性方面，本研究显示，埃克替尼的不良反应以皮疹和腹泻为主，发生率和既往ICOGEN及近期报道^[6, 15]^相似，但低于吉非替尼和厄洛替尼的不良反应发生率^[4-6]^。说明埃克替尼的耐受性较好。

作为回顾性分析，本研究仅仅纳入49例患者，存在样本量较小的问题。另外，目前EGFR突变检测推荐测序法和ARMS（amplification refractory mutation system）两种检测方法，测序法较ARMS方法检测敏感度低，存在一定的假阴性。本研究采用焦磷酸测序法，不排除部分野生型患者存在一定的假阴性的情况，这也可能是本研究野生型患者疗效较好的一个原因。

本研究通过49例EGFR状态明确的患者的疗效分析表明，埃克替尼治疗中国晚期NSCLC疗效明显，且毒副反应多数可耐受，可以作为一种治疗选择。当然，作为刚刚上市不久的新药，埃克替尼还需要进一步通过前瞻性的临床研究探讨该药在突变患者、一线治疗领域及野生型患者等领域的疗效和安全性。
